# An audit of licenced radiological equipment and personnel in Botswana

**DOI:** 10.4102/jcmsa.v2i1.87

**Published:** 2024-09-26

**Authors:** Garebue D. Nlashwa, Morrison Sinvula, Setso O. Setso, Wallace Miller, Molatedi Lesiamang, Tashinga Maboreke, Richard D. Pitcher

**Affiliations:** 1 Division of Radiodiagnosis, Department of Medical Imaging and Clinical Oncology, Faculty of Medicine and Health Sciences, Stellenbosch University, Cape Town, South Africa; 2 Ministry of Health Botswana, Gaborone, Botswana; 3 Department of Radiology, Faculty of Medicine, University of Botswana, Gaborone, Botswana; 4 Department of Radiology, Sir Ketumile Masire Teaching Hospital, Gaborone, Botswana

**Keywords:** Botswana, high- middle-income country, radiologic resources, sparsely populated, public health sector, private healthcare sector

## Abstract

**Background:**

The United Nations encourages national audits of diagnostic imaging equipment and personnel. The World Health Organization (WHO) estimates that 20 X-ray and ultrasound units per million people will meet 90% of global imaging needs. This study assessed registered diagnostic imaging resources in Botswana, a high middle-income, sparsely populated African country.

**Methods:**

Details of registered diagnostic imaging equipment and personnel were extracted from the Botswana Radiation Protection Inspectorate and the Botswana Health Professions Council databases and stratified by imaging modality, professional category, and by geographical region and healthcare sector. Findings were presented as absolute numbers and resources per million people.

**Results:**

Botswana has 130 diagnostic imaging equipment units. General radiography (GR) (*n* = 79) 60%, mammography (*n* = 15; 12%), fluoroscopy (*n* = 13; 10%), computed tomography (*n* = 13; 10%), magnetic resonance (*n* = 6; 5%), digital subtraction angiography (*n* = 3; 2%) and radioisotope (*n* = 1; 0.7%). General radiography is the only modality where overall public sector resources (*n* = 44/79, 56%) exceed those of the private sector. Overall GR meet WHO guidelines, while it exceeded WHO guidelines (42–63 units/106 people) in most sparsely populated districts. There are 171 registered radiation workers; 88% (*n* = 152), radiographers, 9% (*n* = 15) radiologists and 2% (*n* = 4) medical physicists. Fifty three per cent of radiographers (*n* = 80) and 20% of radiologists (*n* = 3) work in the public sector.

**Conclusion:**

This study provides novel insights into the provision of radiological resources to sparsely populated rural communities.

**Contribution:**

The study demonstrates a comprehensive analysis of Radiological resources in an upper-middle-income country in Africa, highlighting important data for medium/long term planning towards achieving an equitable imaging access.

## Introduction

The World Health Organization (WHO) recognises health technology, including diagnostic imaging, as one of the six essential building blocks of any healthcare system.^1,2^ It considers basic radiological services essential for effective primary care^1,3^ and estimates that 20 X-ray and 20 ultrasound units for every million people will meet 90% of global imaging needs.^4,5,6^ However, it is estimated that two-thirds of the world’s population has no access to diagnostic imaging, with the need greatest in rural populations of low- and middle-income countries (LMICs).^4,5^

In May 2007, the 60th United Nations (UN) World Health Assembly adopted Resolution 60.29, urging member states to ‘collect, verify, update and exchange information on health technologies, in particular medical devices, as an aid to their prioritization of needs and allocation of resources’.^6^ However, radiological resources of LMICs remain poorly documented. Data on existing imaging equipment can inform national strategy and policy.^4,5^ Resource disparities can be redressed if such data are available. It is in this context that the Division of Radio diagnosis at Stellenbosch University is evaluating diagnostic radiology resources in African countries, with a view to providing reference data for healthcare planning. To date, analyses have been completed for South Africa (SA), Tanzania, Zimbabwe, Zambia, Kenya, Ghana and Uganda.^3,4,5,7,8,9,10^

However, there has been no detailed evaluation for Botswana. A landlocked southern African country, with an area of 581 730 km^2^, an estimated population of 2 million people and an average population density of 3.5 persons per square kilometre, Botswana is one of the most sparsely populated countries on earth.^11,12^

At the time of independence from Great Britain in 1966, Botswana was the world’s 3rd poorest country.^13^ Following the discovery of diamonds in 1967, it became one of the fastest developing economies, globally, and is hailed as one of Africa’s economic successes. It is now classified as an upper middle-income country based on the World Bank criteria, having increased gross domestic product (GDP) from $51.5 million in 1960 to an estimated $17.6 billion in 2021.^12,14,15,16,17^

Although the country has an impressive governance track record and has enjoyed sustained economic growth, levels of poverty and inequality are high,^17,18,19^ with 16% of the population living in extreme poverty.^12^ Unemployment is approximately 18%, with a youth unemployment of 37%.^1^ The 2020 average Gini coefficient was 0.50, compared to the African average of 0.44, highlighting a paradox of economic growth and inequality.^12,18,19,20^

The prevalence of human immune deficiency virus (HIV) and/or acquired immune deficiency syndrome (AIDS) and tuberculosis (TB) has curtailed economic development of Botswana. The first HIV-infection was reported in 1985, and by 1999, the adult prevalence was 30%.^12,21,22^ However, a successful multi-sectorial response to the pandemic lowered the prevalence to 20% within two decades.^17,20,23^ By 2021, Botswana had exceeded the United Nations Programme on HIV/AIDS (UNAIDS) 95-95-95 targets: with 95% of the population being aware of their HIV status, of whom 98% were on treatment, and 98% virally supressed.^24^

In 2001, African Heads of State committed to allocate at least 15% of government expenditure to health, to address Africa’s substantial disease burden, particularly with respect to HIV and/or AIDS, TB and malaria.^25,26^ By 2005, Botswana was one of the few countries to have met this commitment.^25,26^ However, health allocation has averaged 6% of the national budget over the past decade.^26,27^ Nonetheless, total per capita health expenditure ($483.00 in 2018) is well above most Southern Africa Development Community (SADC) and WHO African Region countries.^12,17,27^ Despite this substantial expenditure, maternal, neonatal and under 5 mortality rates are 166/100 000, 18/1000 and 42/1000, respectively, while United Nations Sustainable Development Goal (SDG) targets for these indicators are 70/100 000, 12/1000 and 25/1000, respectively.^1,12,17^

Botswana is committed to decentralised public health care and universal health coverage. To this end, District Health Management Teams (DHMTs) are currently being developed. The country is divided into 17 administrative districts and has an extensive network of health facilities, including hospitals, clinics, health posts and mobile stops, with primary health care the pillar of the health delivery system. There are 3 national referral hospitals, 7 District General Hospitals, 2 Mine Hospitals and 16 Primary Hospitals. There is a new, partially operational, quaternary facility, the Sir Ketumile Masire Hospital in Gaborone (South-East District). Majority (95%) of the total population and 89% of the rural population live within 8 kilometres of a health facility.^17,20,28^

The University of Botswana (UB) School of Medicine, founded in 2009, has produced more than 300 MBChB graduates.^23^ Six months after the opening of the medical school, residency programmes in internal medicine, paediatrics, public health, anaesthesia, emergency medicine, family medicine and pathology were established. Some programmes used a hybrid system whereby students did part of their training in SA, in line with an agreement between the UB and the Colleges of Medicine of SA (CMSA).^23,30^ By 2018, 40 local doctors had graduated as specialists, 20 by way of the novel hybrid programme.^30^ The UB also offers undergraduate degrees in nursing, pharmacy and medical laboratory sciences. There is no radiography training, with a resultant critical shortage of radiographers. All healthcare professionals are required to register with the Botswana Health Professions Council (BHPC). The Botswana Radiation Protection Inspectorate (BRPI) is responsible for registration of all healthcare equipment utilising ionising radiation.

### Aim

The aim of this study was to conduct a comprehensive audit of licenced Botswana diagnostic imaging resources.

## Research methods and design

This 2021 audit was conducted in collaboration with the BRPI, the Ministry of Health (MoH) and the BHPC. Data on licenced diagnostic imaging equipment were extracted from the BRPI database and included general radiography (GR), fluoroscopy (FL) mammography (MM), computed tomography (CT), digital subtraction angiography (DSA), magnetic resonance imaging (MRI) and radioisotope units. Data on licenced diagnostic imaging personnel were extracted from the BHPC database and included radiologists, radiographers, nuclear physicians and medical physicists. These data were correlated with those of the MoH for personnel currently resident in the country.

Population data were sourced from the preliminary results of the population and housing census of March 2022 and stratified by geographical region.

All data were captured on a customised Microsoft (MS) Excel spread sheet. Equipment and personnel data were stratified by imaging modality and professional category, respectively, and were analysed by geographical region and health care sector.

For the purposes of this study, the urban districts of Gaborone and Lobatse were analysed in conjunction with the South Eastern District, the urban district of Jwaneng with the Southern District, the urban district of Francistown with the North-East District and the administrative districts of Mahalapye, Palapye, Serowe, Sowa Town and Orapa with the Central district (Table 1^5,7,9,10^ and Table 2).

**TABLE 1:** Botswana’s registered radiological equipment resources by modality, district and health care sector per million population.

District	Total population	Area in km^2^	Density in people/km^2^	GR				FL				MM				CT				MRI				DSA				Gamma camera			
				Public	Private	Total	Total/10^6^	Public	Private	Total	Total/10^6^	Public	Private	Total	Total/10^6^	Public	Private	Total	Total/10^6^	Public	Private	Total	Total/10^6^	Public	Private	Total	Total/10^6^	Public	Private	Total	Total/10^6^
Kweneng district	387 983	31 100	12	7	3	10	26	0	1	1	3	0	2	2	5	0	1	1	3	0	1	1	3	0	1	1	3	0	1	1	3
South Eastern district (including Gaborone and Lobatse)	387 537	1991	194	8	18	26	67	2	5	7	18	2	7	9	23	2	4	6	15	1	3	4	10	0	1	1	3	0	-	-	-
North Eastern district (including Francistown)	172 776	5199	33	4	3	7	41	1	3	4	23	-	3	4	23	1	3	4	23	0	1	1	7	0	1	1	7	0	-	-	-
Central district (including Selebi Phikwe and Sowa)	706 448	142 277	5	11	6	17	24	0	1	1	1.4	0	-	-	-	1	0	1	1.4	0	-	-	-	0	-	-	-	0	-	-	-
Ngamiland district	198 436	109 130	1.8	4	4	8	40	0	-	-	-	0	-	-	-	1	0	1	5	0	-	-	-	0	-	-	-	0	-	-	-
Southern district (including Jwaneng)	240 752	28 470	9	3	1	4	16	0	-	-	-	0	-	-	-	0	-	-	-	0	-	-	-	0	-	-	-	0	-	-	-
Chobe district	28 743	20 800	1.4	1	0	1	35	0	-	-	-	0	-	-	-	0	-	-	-	0	-	-	-	0	-	-	-	0	-	-	-
Ghanzi district	56 067	117 910	0.5	2	0	2	35	0	-	-	-	0	-	-	-	0	-	-	-	0	-	-	-	0	-	-	-	0	-	-	-
Kgalagadi district	57 758	105 200	0.6	3	0	3	52	0	-	-	-	0	-	-	-	0	-	-	-	0	-	-	-	0	-	-	-	0	-	-	-
Kgatleng district	121 882	7920	15	1	0	1	8	0	-	-	-	0	-	-	-	0	-	-	-	0	-	-	-	0	-	-	-	0	-	-	-

**Total**	**2 358 382**	**570 166**	**4**	**44**	**35**	**79**	**33**	**3**	**10**	**13**	**6**	**3**	**12**	**15**	**6**	**5**	**8**	**13**	**6**	**1**	**5**	**6**	**3**	**0**	**3**	**3**	**1.3**	**0**	**1**	**1**	**0.4**

Note: Please see the full reference list of the article Nlashwa GD, Sinvula M, Setso SO, et al. An audit of licenced radiological equipment and personnel in Botswana. J Coll Med S Afr. 2024;2(1), a87. https://doi.org/10.4102/jcmsa.v2i1.87, for more information. Private: public ratio: GR = 1:1.25; FL = 03:01; MM = 04:01; CT = 1.6:1; MRI = 05:01; DSA and Gamma Camera = n/a.

GR, general radiography; FL, fluoroscopy; MM, mammography; CT, computed tomography; DSA, digital subtraction angiography; MRI, magnetic resonance imaging.

**TABLE 2:** Botswana’s registered radiographers by district, health care sector per million population.

District	Public (*n*)	Private (*n*)	Total	
			*N*	*n* †
Kweneng district	6	7	13	34
South Eastern district (including Gaborone and Lobatse)	20	35	55	142
North Eastern district (including Francistown)	13	8	21	122
Central district (including Selebi Phikwe and Sowa)	25	7	32	45
Ngamiland district	5	1	6	30
Southern district (including Jwaneng)	3	3	6	25
Chobe district	2	2	4	70
Ghanzi district	1	0	1	18
Kgalagadi district	4	0	4	69
Kgatleng district	2	0	2	16

**Total**	**81**	**63**	**144**	**61**

Note: Sourced from BHPC, correlated with information from the MoH exclusive of registered radiographers (*n* = 4) in administrative positions.

BHPC, Botswana Health Professions Council; MoH, Ministry of Health.

† , per 10^6^.

Resources were quantified by absolute number and the number per million people. Healthcare sector analyses assumed 17% of the population have access to private medical insurance.^28,29^

Data were compared with those of SA, Tanzania, Zimbabwe, Zambia, Uganda, Kenya and Ghana.^3,4,5,7,8,9,10^

### Ethical considerations

Ethical approval to conduct this study was obtained from the Stellenbosch University Faculty of Medicine and Health Sciences Health Research Ethics Committee (No. S21/10/204), the University of Botswana Office of Research Development and the Botswana Ministry of Health Research Unit.

## Results

### Registered diagnostic imaging equipment

#### Overview

There are 130 licenced diagnostic imaging units in Botswana (Table 1). General radiography units (*n* = 79) represent 60% of all equipment, followed by MM (*n* = 15; 12%), FL (*n* = 13; 10%), computed tomography (CT) (*n* = 13; 10%), MRI (*n* = 6; 5%), DSA (*n* = 3; 2%) and radioisotope units (*n* = 1; 0.7%). There are 34 GR units, 6 MM/FL/CT units, 2 MRI units and a single DSA unit per million people, with 6 GR units per CT scanner (79 vs. 13) and 16 GR units per MRI scanner (79 vs 5). General radiography resources are well above the minimum WHO requirement of 20 units per million people.

#### Analysis by geographic area

**FIGURE 1:**
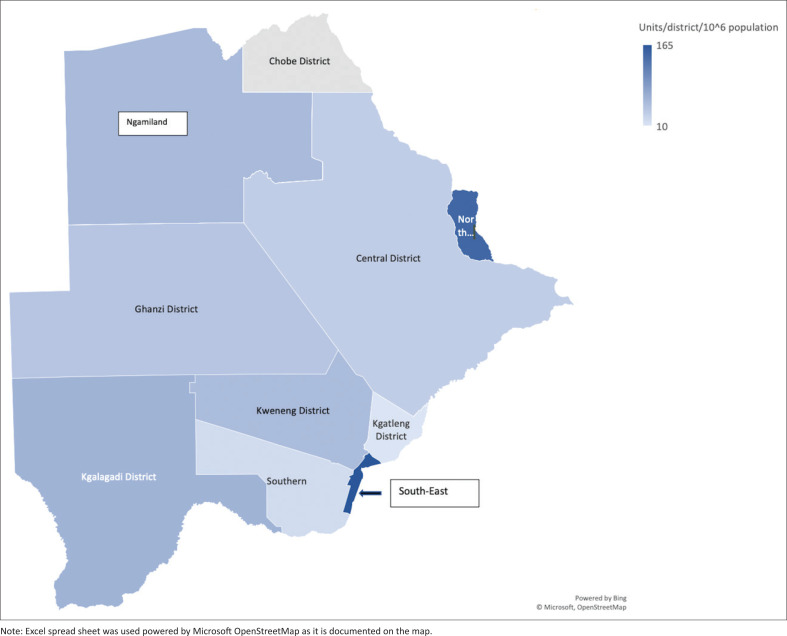
Map of Botswana displaying the total number of diagnostic units in the country per million populations.

Only one district (Kweneng) has a full complement of radiological resources, including GR, FL, MM, CT, MRI, DSA and radioisotope imaging (Table 1 and Figure 1). Five districts (*n* = 5) have direct access to CT, 4 to FL and 3 to MRI. All those with access to FL and MRI also have direct CT access. Half the districts (*n* = 5) have access to only GR.

More than half of all equipment units (*n* = 70, 54%) are located in two districts in the south-east of the country (South East, Kweneng). These districts have a combined land area of approximately 33 000 km^2^, being 6% of the total land area and a combined population of 775 475 people or approximately one-third (32.8%) of the national population.

#### Analysis by modality

*General radiography*: General radiography resources are the most numerous (*n* = 79/130) and widely distributed, with public sector resources available in all geographic areas and private sector resources in six districts. General radiography is the only modality where overall public sector resources (*n* = 44/79, 56%) exceed those in the private sector (*n* = 35/79, 44%). General radiography has the lowest discrepancy (4:1) between public and private resources by million people. Of the 10 districts, 7 meet the WHO recommendation of 20 public sector GR units per million people, while the Central (19 units/106 people), Southern (15 units/106 people) and Kgatleng districts (10 units/106 people) are slightly below this threshold.

*Fluoroscopy*: Fluoroscopy units represent just 10% (*n* = 13/130) of resources. Less than a third of the units (*n* = 3/10, 30%) are in the public sector, and these are confined to the major urban areas of Gaborone and Francistown. Eight of the 10 districts thus have no direct fluoroscopic access.

*Mammography*: Mammography units constitute 12% (*n* = 15/130) of equipment resources, with only one-fifth (*n* = 3/15) in the public sector. Geographic distribution is comparable to FL, with public sector units confined to Gaborone and Francistown.

*Computed tomography*: Computed tomography units represent 10% (*n* = 13/130) of all resources, with almost 40% (*n* = 5/13, 38%) in the public sector. Computed tomography is the only public sector modality other than GR that is available outside of Gaborone and Francistown, with units in Maun and Mahalapye. There is 1 CT scanner for every 9 GR units in the public sector. The private sector has 62% (*n* = 8) of units nationally, equivalent to 20 units/106 people, exceeding public sector resources six-fold (3 units/106 people).

*Magnetic resonance imaging*: MRI resources represent 5% (*n* = 6/130) of resources, with just 16% (*n* = 1/6) in the public sector. The only public sector unit is in Gaborone.

#### Registered diagnostic imaging personnel

There are 164 registered radiation workers excluding administrative personnel, with diagnostic radiographers (*n* = 144) constituting 88.0%, radiologists (*n* = 15) 9.0% and medical physicists (*n* = 4) 2.5% (Table 2).

Just over half the radiographers (*n* = 81; 52%) are in the public sector, and almost half (*n* = 68; 47%) are in just two districts in the southeast of the country (South East, Kweneng). The geographic distribution of radiographers is reflected in Table 2. One-fifth of radiologists (*n* = 4) are in the public sector, 1 at the UB and 10 in the private sector. All medical physicists (*n* = 4; 100%) are in the private sector. There are no data on the geographic distribution of radiologists or medical physicists. There are no registered sonographers in Botswana.

The number of radiographers, radiologists and medical physicist per million population is 61, 6.4 and 1.8, respectively.

#### Comparison with published African radiological resources

Comparisons with key economic, healthcare and radiological data for Uganda, Tanzania, Zimbabwe, Zambia, Kenya, Ghana and SA are reflected in Table 3.^3,4,5,6,7,8,9,10^ Of note, Botswana has the smallest population and is the most sparsely populated country of those reviewed. Also noteworthy is that Botswana is the second higher middle-income country to be reviewed, after SA. Botswana has the highest GDP per capita ($7347.00) and the second highest (after SA) health expenditure per capita ($481.00). Its radiological equipment resources are comparable with, or exceed, the best available among the countries reviewed.

**TABLE 3:** Comparison of Botswanan health/economic indicators and imaging resources with other African countries.

Variable	Uganda (low income)	Zimbabwe (low income)	Tanzania* (low income)	Zambia (low income)	Kenya (lower middle-income)	Ghana (lower middle-income)	South Africa† (upper middle-income)	Botswana (upper middle-income)
**Demographics**								
Area (× 10^3^ km^2^)	241.00	390.00	890.10	753.00	583.00	238.50	1219.10	582.00
Population (× 10^6^)	45.70	14.90	597.90	12.60	54.90	31.40	59.20	2.30
Population density (people/km^2^)	235.00	39.00	69.00	25.00	97.00	140.00	49.00	4.00
GDP (10^9^ USD)	40.30	26.20	48.06	21.20	95.50	77.60	350.14	17.61
GDP per capita (current USD)	920.00	1737.00	1135.00	1120.00	2081.00	6178.00	6994.00	7347.60
% GDP spent on health	3.30	7.70	3.80	5.31	4.59	3.99	9.10	6.05
Health expenditure per capita (USD)	43.14	103.03	49.00	69.00	208.00	193.00	593.00	481.53
% budget allocated to health	12.00	10.00	7.00	5.00	6.50	8.00	14.00	11.00
% of total population insured	5.00	6.00	16.60	3.00	19.00	59.00	17.00	17.00
**Healthcare indicators**								
Life expectancy	64.00	61.00	66.00	64.00	67.00	64.00	64.00	70.00
Maternal mortality ratio per 100 000 live births	375.00	458.00	524.00	224.00	342.00	334.00	88.00	166.00
Under 5 years mortality rate per 1000 live births	43.00	54.00	49.00	61.00	42.00	45.00	35.00	45.00
Death rate (crude per 1000 people)	6.00	8.00	6.00	6.00	5.00	7.00	9.00	6.00
HIV prevalence	5.40	11.90	4.70	11.00	4.00	1.62	19.00	19.90
TB incidence per 100 000	196.00	193.00	222.00	319.00	251.00	440.00	554.00	236.00
**Public sector radiology equipment (units/10^6^ people)**								
GR	2.00	11.00	5.70	11.00	9.00	8.00	19.80	23.00
FL	0.30	0.10	0.83	0.60	2.10	0.60	2.50	2.00
MM	0.08	0.20	0.24	1.00	1.30	0.60	1.30	2.00
CT	0.20	0.60	0.08	0.50	1.30	0.70	1.70	3.00
MRI	N/S	0.20	0.05	0.10	N/S	0.30	0.30	0.50

*Source:* Kiguli-Malwadde E, Byanyima R, Kawooya MG, Mubuuke AG, Basiimwa RC, Pitcher RC. An audit of registered Ugandan radiology equipment resources. Pan Afr Med J. 2020;37(295):1–12. https://doi.org/10.11604/pamj.2020.37.295.22046; Maboreke T, Banhwa J, Pitcher RD. An audit of licensed Zimbabwean radiology equipment resources as measure of healthcare access and equity. Pan Afr Med J. 2019;34:1–10. https://doi.org/10.11604/pamj.2019.34.60.18935; Ngoya PS, Muhogora WE, Pitcher RD. Defining the diagnostic divide: An analysis of registered radiological equipment resources in a low-income African country. Pan Afr Med J. 2016;25:99. https://doi.org/10.11604/pamj.2016.25.99.9736; Mapuranga H, Pitcher RD, Jakanani GC, Banhwa J. An audit of Zimbabwean public sector diagnostic ultrasound services. Pan Afr Med J. 2021;39:99. https://doi.org/10.11604/pamj.2021.39.99.28342; Mbewe C, Chanda-Kapata P, Sunkutu-Sichizya V, et al. An audit of licenced Zambian diagnostic imaging equipment and personnel. Pan Afr Med J. 2020;36:1–14. https://doi.org/10.11604/pamj.2020.36.32.21043; Kabongo JM, Nel S, Pitcher RD. Analysis of licensed South African diagnostic imaging equipment. Pan Afr Med J. 2015;22:1–9. https://doi.org/10.11604/pamj.2015.22.57.7016; Gathuru LM, Elias GDO, Pitcher RD. Analysis of registered radiological equipment in Kenya. Pan Afr Med J. 2021;40:205; Bour BK, Sosu EK, Hasford F, et al. National inventory of authorized diagnostic imaging equipment in Ghana: Data as of September 2020. Pan Afr Med J. 2022;41:301. https://doi.org/10.11604/pamj.2022.41.301.30635

† , Countries with the highest GDP.

GDP, gross domestic product; USD, United States dollar; HIV, human immune deficiency virus; TB, tuberculosis; GR, general radiography; FL, fluoroscopy; MM, mammography; CT, computed tomography; MRI, magnetic resonance imaging; N/S, not specified.

## Discussion

This is the first comprehensive audit of diagnostic imaging resources in Botswana. The study provides important insights into the provision of radiological resources in a sparsely populated country. With a population density of 3.7 people/km^2^, Botswana is the most sparsely populated of the eight African countries where audits of radiological equipment resources have been conducted to date. Botswana also has the fourth lowest population density of all African countries, after Namibia (3.2/km^2^), Mauritania (3.4/km^2^) and Libya (3.6/km^2^).^31^ The challenge of radiological resource provision is compounded by Botswana’s unequal population distribution, with almost half the population (*n* = 1 097 226; 47%) concentrated in four districts in the southeast of the country (South East, Southern, Kweneng and Kgatleng), which have a combined land area of 69 481 km^2^ or 12% of the total land area. Figure 1 displays the distribution of diagnostic equipment’s across the country.

The key finding of this study is Botswana’s commitment to the provision of accessible basic public sector radiological services. This is evidenced by public sector GR units being available in all 10 geographical regions analysed, with 7 of the 10 regions meeting or exceeding the WHO recommendation of 20 units per million people. Furthermore, one region (Central district) falls just short (19 units/106 people) and could be construed as effectively meeting the target. It is noteworthy that the most sparsely populated regions, being Ghanzi (0.5 people/km^2^), Kgalagadi (0.6/km^2^) and Chobe (1.4/km^2^) have the highest basic resources, with 43, 63 and 42 GR units/106 people, respectively. This represents a clear undertaking on the part of Botswana health authorities to compensate for the low population density by the provision of resources that far exceed the WHO guidelines.

This study provides important data for short and/or medium-term strategic planning for radiological resources in Botswana. It has shown a shortfall in GR resources in Kgatleng, which currently has 10 units/106 people and a population of 100 000 people. The provision of just one additional GR unit would meet WHO guidelines. A shortfall also exists in the Southern District, with 15 units/106 people and a population of approximately 200 000 people. The provision of one additional unit would thus allow the region to meet WHO recommendations. The provision of just two additional GR units would allow all geographical regions to meet WHO guidelines for basic radiological services. The ratio of one CT scanner for every nine GR units in the Botswana public sector is also broadly aligned with WHO guidelines, which suggest that 90% of all diagnostic imaging requirements can be met by the provision of one X-ray and ultrasound unit for every 50 000 people. Conversely, the WHO suggests that only 10% of diagnostic imaging needs involve more sophisticated imaging modalities such as CT and MRI. A GR:CT ratio of 9:1 would thus appear to be appropriate.

Although the country has made notable strides with respect to imaging equipment, there remains a substantial deficit in imaging personnel, with the number of radiographers and radiologists per million people being 61 and 6.4, respectively. These numbers are approximately half that of SA, also a high middle-income country, which has 123 radiographers and 17 radiologists per million people. Both Botswana and SA have considerably lower radiologist resources per million people than high-income countries such as the United States (*n* = 100), Australia (*n* = 87) and the United Kingdom (*n* = 56).^32^

This study is only the second of its kind involving an upper middle-income African country. It thus contributes to a better understanding of how economic parameters impact the provision of radiological resources on the continent. The findings are intuitive and have shown that resources are broadly aligned with per capita GDP and health expenditure and thus with World Bank stratification of countries by income.

Strengths of this study include analyses of both equipment and personnel resources. This is only the second study of African radiological resources to include data on imaging personnel. The Zambian analysis by Mbewe et al. was the only previous study to include such data. It is strongly recommended that all future studies of national diagnostic imaging resources include such dual analyses.

An additional major strength is its contribution to the discourse on the provision of radiological resources to sparsely populated regions. The finding that GR resources in districts with low population density are more than double the recommended WHO resources for basic radiological services could inform international norms for such regions. The WHO may thus consider modifying its recommendations to accommodate geographical regions with population densities approximating one person per square kilometre.

### Limitations

A limitation of this study, which is common to all analyses of registered diagnostic imaging equipment, was the exclusion of ultrasound equipment. Ultrasound does not emit ionising radiation and is thus exempt from registration with a regulatory authority. Accurate data on the number of diagnostic ultrasound units are not thus available in most national inventories in African countries. Furthermore, this is a quantitative analysis and does not necessarily reflect equipment functionality. A further limitation was the absence of data on the geographic distribution of radiologists and medical physicists.

Going forward, the ideal would be for national regulatory authorities in African countries to publish annual updates of registered diagnostic imaging resources, including analyses of functionality. This would provide ongoing accurate data on equity in access to diagnostic imaging. Consideration should also be given to the registration of diagnostic ultrasound equipment to address the dearth of data in this domain.

## Conclusion

This study provides novel insights into the provision of radiological resources to sparsely populated rural communities.

### What is known about the topic

This study is aligned with six previous publications, which documented the registered radiological resources in SA, Zimbabwe, Zambia, Kenya, Uganda and Ghana.

### What this study adds

The study demonstrates a comprehensive analysis of radiological resources in an upper-middle-income but sparsely populated country. It further highlights resource disparities between the private and public sectors, important data for medium and/or long-term planning towards achieving an equitable imaging access.
