# Corrigendum: An audit of licenced radiological equipment and personnel in Botswana

**DOI:** 10.4102/jcmsa.v2i1.152

**Published:** 2024-11-15

**Authors:** Garebue D. Nlashwa, Morrison Sinvula, Setso O. Setso, Wallace Miller, Molatedi Lesiamang, Tashinga Maboreke, Richard D. Pitcher

**Affiliations:** 1 Division of Radiodiagnosis, Department of Medical Imaging and Clinical Oncology, Faculty of Medicine and Health Sciences, Stellenbosch University, Cape Town, South Africa; 2 Ministry of Health Botswana, Gaborone, Botswana; 3 Department of Radiology, Faculty of Medicine, University of Botswana, Gaborone, Botswana; 4 Department of Radiology, Sir Ketumile Masire Teaching Hospital, Gaborone, Botswana

In the published article, Nlashwa GD, Sinvula M, Setso SO, et al. An audit of licenced radiological equipment and personnel in Botswana. J Coll Med S Afr. 2024;2(1), a87. https://doi.org/10.4102/jcmsa.v2i1.87 on page 5, the following statistical data was provided:

Instead of:

One-fifth of radiologists (*n* = 4) are in the public sector

It should be:

One-fifth of radiologists (*n* = 3) are in the public sector

The authors apologise for this error. The correction does not change the study’s findings of significance or overall interpretation of the study’s results or the scientific conclusions of the article in any way.

